# Single-Cell Point Constrictions for Reagent-Free High-Throughput Mechanical Lysis and Intact Nuclei Isolation

**DOI:** 10.3390/mi10070488

**Published:** 2019-07-19

**Authors:** Xiaomin Huang, Xiaoxing Xing, Chun Ning Ng, Levent Yobas

**Affiliations:** 1 Department of Electronic and Computer Engineering, The Hong Kong University of Science and Technology, Clear Water Bay, Kowloon, Hong Kong, China; 2 College of Information Science and Technology, Beijing University of Chemical Technology, Beijing 100029, China; 3 Division of Biomedical Engineering, The Hong Kong University of Science and Technology, Clear Water Bay, Kowloon, Hong Kong, China

**Keywords:** cell lysis, constriction, DNA, protein, microfluidic, nuclei

## Abstract

Highly localized (point) constrictions featuring a round geometry with ultra-sharp edges in silicon have been demonstrated for the reagent-free continuous-flow rapid mechanical lysis of mammalian cells on a single-cell basis. Silicon point constrictions, robust structures formed by a single-step dry etching process, are arranged in a cascade along microfluidic channels and can effectively rupture cells delivered in a pressure-driven flow. The influence of the constriction size and count on the lysis performance is presented for fibroblasts in reference to total protein, DNA, and intact nuclei levels in the lysates evaluated by biochemical and fluoremetric assays and flow-cytometric analyses. Protein and DNA levels obtained from an eight-constriction treatment match or surpass those from a chemical method. More importantly, many intact nuclei are found in the lysates with a relatively high nuclei-isolation efficiency from a four-constriction treatment. Point constrictions and their role in rapid reagent-free disruption of the plasma membrane could have implications for integrated sample preparation in future lab-on-a-chip systems.

## 1. Introduction

Cell lysis—the process of breaking open cells by disrupting the cytoplasmic membrane to release the cellular content—is the first step in “sample preparation”, whether it be for the extraction and purification of nucleic acids or proteins or the fractionation of subcellular organelles (e.g., mitochondria). Considered as a gateway to a myriad of downstream processes in various fields, including molecular biology, drug discovery, immunogenetics, and clinical diagnostics, cell lysis is achieved more often through biochemical agents (e.g., detergents, enzymes, bacteriophages) than physical means, including a fluidic shear and an electric or thermal shock [1,2,3]. Subsequent purification is crucial for the removal of lytic agents known to inhibit downstream processes and yet adds complexity to the process due to repetitive cycles of washing and elution (i.e., centrifugation and manual pipetting). Therefore, automating the “sample preparation” without compromising the quantity (yield), as well as the quality (undenatured state), is desired to address the bottleneck issue in accessing subcellular targets. To this end, researchers have turned to microfluidic systems and investigated various forms of on-chip cell lysis for obtaining a fully integrated lab-on-a-chip sample preparation.

Microfluidic approaches to cell lysis, at first, adopted the miniaturization of conventional methods, many of which have benefited from scaling laws in regards to reduced diffusion lengths and processing times, and lowered operating voltages. Sethu et al., selectively lysed erythrocytes by creating a focused blood stream flanked on both sides by lysis buffer [4]. Morton et al., lysed cells by moving them in a bump array across streamlines carrying lysis and wash buffers [5]. Lu et al., electroporated cells irreversibly, disrupting them in between saw-tooth microelectrodes [6]. Others placed wire electrodes in reservoirs and ruptured cells with constriction under an intensified field strength, thereby avoiding the issues with microelectrodes due to electrolysis (e.g., metal degradation, extreme pH, bubbles) [7,8]. Interestingly enough, hydroxide ions generated at the microelectrodes (electrolysis) were later exploited to lyse cells, negating the requirement of a high-voltage supply and the injection and removal of a lytic agent (hydroxides become quenched by recombination with protons generated at the anode) [9]. Privorotskaya et al., thermally lysed fibroblasts on microcantilever heaters [10]. Ke et al., disrupted bacteria in a thermally-isolated microchamber [11]. Apart from resistive heating, researchers pursued wireless heating (induction or plasmonics) to rupture cells in simplified chip designs [12,13]. Indirect heating and cavitation-induced shock waves either by sonication [14] or a pulsed laser beam [15,16] were also utilized to disrupt bacterial spores and cells. Collisions with particles (bead beating) on a spinning platform [17,18] and compression under a deforming diaphragm [19] were shown to be effective for mechanically rupturing cells. While each of these approaches has its own merits, limitations do exist, including the denaturation of proteins due to excessive heat or pH, a low throughput, a tight operating window, and the requirement of reagents or special hardware operated by a skilled user.

Sharp micro/nanostructures built into the flow path deliver direct mechanical insult to cells and have been pursued by several groups for reagent-free cell lysis to address some of those limitations. Lee and colleagues utilized narrow passages to squeeze cells in a pressure-driven flow, reporting a considerable rise in protein accessibility for passages with knife-like sharp edges as opposed to smooth sidewalls [20]. Kim et al., pushed cells through an array of microposts decorated with nanowires protruding sharp tips and analyzed the lysate for the protein and nucleic acid yield [21]. Others demonstrated chips operated by a manually driven syringe, drawing attention to the equipment-free nature of the approach. Yun et al., developed a handheld unit housing a silicon chip in which cells are pressed against sharp edges of microposts; the authors found that the protein levels were comparable to those obtained from a conventional method [22]. Likewise, So et al., showed a handheld unit sandwiching a porous silicon membrane with nanospikes protruding out on the surface and achieved comparable or higher protein and nucleic acid recovery than obtained by conventional methods [23]. The authors subsequently improved the membrane by replacing the nanospikes with nanowires, followed by a silica surface coating for nucleic acid binding [24]. Although all these studies have raised considerable interest, they have continued to treat cells as a population. Microfluidics, however, has the capacity to treat cells discretely in a highly rapid serial fashion, as demonstrated by the studies on cell electroporation [25] and mechanoporation [26]. This is an important feature for the isolation of intact sub-cellular organelles like nuclei.

**Figure 1:**
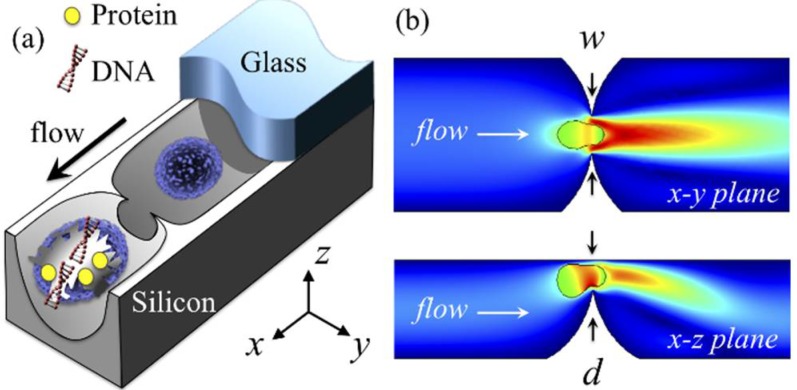
Single-cell point constriction for reagent-free cell lysis. (**a**) 3D rendering: cell being ruptured by the ultra-sharp edge of a round constriction. (**b**) Cutaway views: cell undergoing excessive rapid deformation through a point constriction. Colour spectrum: flow velocity (increasing from blue to red). The glass cover is partially illustrated for clarity.

Here, we introduce a silicon-glass device featuring point constrictions for reagent-free mechanical cell lysis and intact nuclei isolation on a single-cell basis in continuous flow. Previously, we demonstrated point constrictions for cytosolic delivery, mainly through reversible cell poration arising from rapid membrane deformation [27]. Driving the cell deformation to the extreme leads to irreparable poration or membrane rupture. The concept is described in Figure 1. Point constrictions feature a round shape that is highly localized such that it projects an ultra-sharp rim (a concave “nanoblade”). This nanometer-scale sharpness is a result of a single-step dry-etch process in which opposing etch fronts are directed to merge at a focal point. By varying the constriction size and count, we present the total protein and nucleic acid concentrations obtained from device treatments in comparison to those resulting from a conventional chemical method. Notably distinct from other studies, device lysates contain many intact nuclei, which we attribute to the concave nanoblades possibly shaving off the cell membrane and cytoskeleton and/or to the excess cell deformation pitting out the intact nucleus. We present the intact nuclei isolation efficiency based on the flow cytometric analyses. This work is one of the rare studies on the topic of nuclei isolation on a microfluidic chip [28,29], and, to the best of our knowledge, is the first account to achieve this task based on mechanical means.

## 2. Materials and Methods

### 2.1. Device Fabrication

Silicon wafers that were 100 mm in diameter and double-sided polished and a 3 μm thick oxide cover were submitted to a two-mask process (Figure S1). Briefly, the fluidic layout was outlined in the front oxide layer through photolithography, followed by an advanced oxide etch; the point constriction sites were marked by introducing discontinuities along the channel patterns. Exposed silicon was removed by an isotropic etch (130 sccm SF_6_, 14 sccm O_2_, 94 m Torr, coil/platen power: 800/11 W), forming the channels as well as the constrictions; the latter emerged as a result of etch fronts laterally advancing and undercutting the oxide film. The size and shape of the constrictions were determined by the total etch time and layout, specifically the corner angles defining the discontinuities, with a value of 90°, and their separation, with a value of 16 μm (Figure S1a). The front oxide was subsequently stripped off in buffered oxide etchant while protecting the wafer backside. A glass wafer was secured in place, enclosing the channels through anodic bonding (800 V, 420 °C, and 600 mbar). The fluidic ports were structured on the wafer backside subsequent to photolithography through an advanced oxide etch and silicon deep reactive ion etch. The photoresist layer was stripped off and the devices were singulated by wafer dicing.

### 2.2. Device Preparation

A custom designed stainless-steel holder featuring o-ring seals was utilized to clamp the device being tested after aligning the respective device reservoirs to the inlet/outlet ports (Figure S2). Nanoport connectors (IDEX Health & Science, Oak Harbor, WA, USA) were used to secure 1/16” OD PTFE tubing (1/32” ID) into the ports. The device was flushed with 5% (*w*/*v*) bovine serum albumin (BSA) in phosphate buffered saline (PBS) solution to block the surface with BSA against the non-specific adsorption of cells and cellular proteins and molecules. Free BSA was then removed by copious washing with PBS. The sample was supplied by a 15 mL Falcon tube with a modified cap (Elveflow, Paris, France) and pressurized from a nitrogen tank (at 7 bar). A further 15 mL Falcon tube was used to collect the lysate, which was subsequently kept on ice for no more than 30 min. Figure S2 also shows the experimental setup.

### 2.3. Cells

Mouse embryo fibroblasts (NIH/3T3) and human colorectal carcinoma cells (HCT116) were cultured in 100 mm dishes in an incubator (with 5% CO_2_ and at 37 °C). As the culture medium, Dulbecco’s modified Eagle’s medium (DMEM) was used with a supplement of 10% fetal bovine serum (FBS) and 1% penicillin/streptomycin. Before each experiment, cells were washed with PBS and treated with trypsin/ethylenediaminetetraacetic acid (EDTA) 0.25% for 2 min. After cells were detached, 5 mL DMEM was added to the culture dish to stop digestion. Detached cells were centrifuged at 1500 rpm for 2 min and re-suspended in DMEM. Unlike NIH/3T3 and HCT116 cells, human chronic myelogenous leukemia cells (K562) were grown in suspension in a cell culture flask. Roswell Park Memorial Institute medium (RPMI) with 10% FBS and antibiotic-antimycotic (1×) served as the culture medium; cell suspensions were centrifuged at 1500 rpm for 2 min and re-suspended in the RPMI before each experiment.

### 2.4. Cell Treatment

The cells harvested were centrifuged at 1000 rpm for 5 min and then resuspended either in PBS or 1× passive lysis buffer (Promega, Madison, WI, USA) supplemented with a protease inhibitor cocktail (Roche, Indianapolis, IN, USA). The volume of the suspension was set to bring the cell population density to, unless otherwise stated, 2.4 × 10^6^ cells/mL. The lysate was incubated on ice for 30 min and the cell suspension in PBS was divided into two fractions of equal volume for device treatment, as well as the control. The control did not undergo any treatment, but incubation for a period of time equivalent to that of the treatment. All the treated and untreated cell suspensions were centrifuged at 6000 × g at 4 °C for 10 min to remove cells (the control) and the cell membrane debris; the supernatants collected were immediately assayed to quantify the total protein and DNA levels. The control results were used as the baseline and subtracted from the treatment results (mechanical and chemical) to disregard cell lysis irrelevant to the treatment.

For the nuclei isolation, the cells harvested were incubated with 5 μM lipophilic carbocyanine membrane dye, DiOC18(3) (DiO), for 15 min at 37 °C. Stained cells were centrifuged thrice at 1500 rpm at 20 °C for 5 min to remove free dye and then resuspended either in PBS (for device treatment) or a lysis buffer containing a non-ionic detergent (10 mM Tris-HCl pH 7.4, 10 mM NaCl, 3 mM MgCl_2_, and 0.5% Triton X-100) at a population density of 1 × 10^6^ cells/mL. Propidium iodide (PI, Sigma-Aldrich, St. Louis, MO, USA) was added at 50 μg/mL to stain the nuclei. After a 5 min incubation period on ice, the lysate was centrifuged at 500 × g for 5 min. The nuclear pellet was resuspended in PBS and sent, along with the lysate collected from the device (without further processing), to a flow cytometer and a confocal microscope to count and inspect the isolated nuclei.

### 2.5. Microscopy

Confocal images were taken on a laser scanning confocal microscope (STED TCS SP5 II, Leica, Wetzlar, Germany) with a 63 × oil-immersion lens; a 20 μL fraction of the lysate was mounted on a glass slide and covered by a coverslip. Images of PI (red laser Ex/Em: 561/640 nm) and DiO (Argon laser Ex/ Em: 488/530 nm) were then captured.

### 2.6. Protein Yield

The total protein content in each sample was obtained from the bicinchoninic acid (BCA) assay (Pierce™ BCA protein assay kit; Thermo Fisher Scientific, Waltham, MA, USA). The assay exploits the well-known biuret reaction, which includes the reduction of Cu^2+^ to a cuprous cation (Cu^+^) in the presence of proteins in an alkaline medium. The chelation of two molecules of BCA with one cuprous ion yields a violet-colored complex, which exhibits strong absorbance at 562 nm in a nearly linear relation with the protein concentration over a broad range (20–2000 μg/mL). Standards were prepared by serially diluting 2 mg/mL BSA with PBS. In total, 25 μL of each standard and lysate obtained chemically or mechanically, as well as the control, were pipetted into microplate wells in replicates of two, with each containing 200 μL of BCA reagent (prepared by mixing the supplied reagents according to the manufacturer’s instructions). The microplate was then put on a plate shaker for 30 s and incubated at 37 °C for 30 min. After a cooling down period, the absorbance at 562 nm was measured from each well thrice using a plate reader (EnVision 2104 Multilabel Plate Reader, PerkinElmer, Waltham, MA, USA). A standard curve was obtained by plotting the BSA concentration against the corrected average measurement of each standard by subtracting the average measurement of the blank (PBS without BSA). For each lysate, the total protein concentration was determined in relation to that of the control (baseline) through quadratic curve fitting.

### 2.7. DNA Yield

The DNA concentration in each sample was quantified using a Quant-iT™ dsDNA high-sensitivity assay (Thermo Fisher Scientific). The assay reagent selectively binds to dsDNA and, upon binding, fluoresces with an intensity linear to the DNA amount in the range of 0.2–100 ng. A total of 10 μL of each λ DNA standard and lysate obtained chemically or mechanically, as well as the control, were pipetted into microplate wells in replicates of two, with each containing 200 μL of the working solution (prepared by mixing the supplied reagent and dilution buffer at a 1:200 *v*/*v* ratio). The microplate was then put on a plate shaker for mixing for 5 min. The fluorescence (ex/em 480/530 nm) of each well was measured thrice using a plate reader (EnVision 2104 Multilabel Plate Reader, PerkinElmer, Waltham, MA, USA). A linear standard curve was obtained by plotting the λ DNA concentration against the average reading of each standard once corrected for that of the blank (without λ DNA). For each lysate, the total DNA concentration was determined in relation to that of the control (baseline) through line fitting.

### 2.8. Flow Cytometry

Lysates, along with the control, were screened for nuclei extraction efficiency using a flow cytometer (Becton Dickinson, FACSAria IIIu, BD Biosciences, San Jose, CA, USA), with data being collected and analyzed using the software FACSDiva (BD Biosciences) and FlowJo (Tree Star Inc., Ashland, OR, USA), respectively. For each sample, a total of 10,000 events were screened and the average event rate was noted based on the total screening time. Fluorophores were excited by 405, 488, and 561 nm lasers. Filters 450/40, 530/30, and 610/20 were applied for the detection of DiO and PI, respectively. A PI intensity gate was applied to the event histograms of the lysates, as well as the control. PI-positive events were identified as the isolated nuclei.

## 3. Results

### 3.1. Device

**Figure 2:**
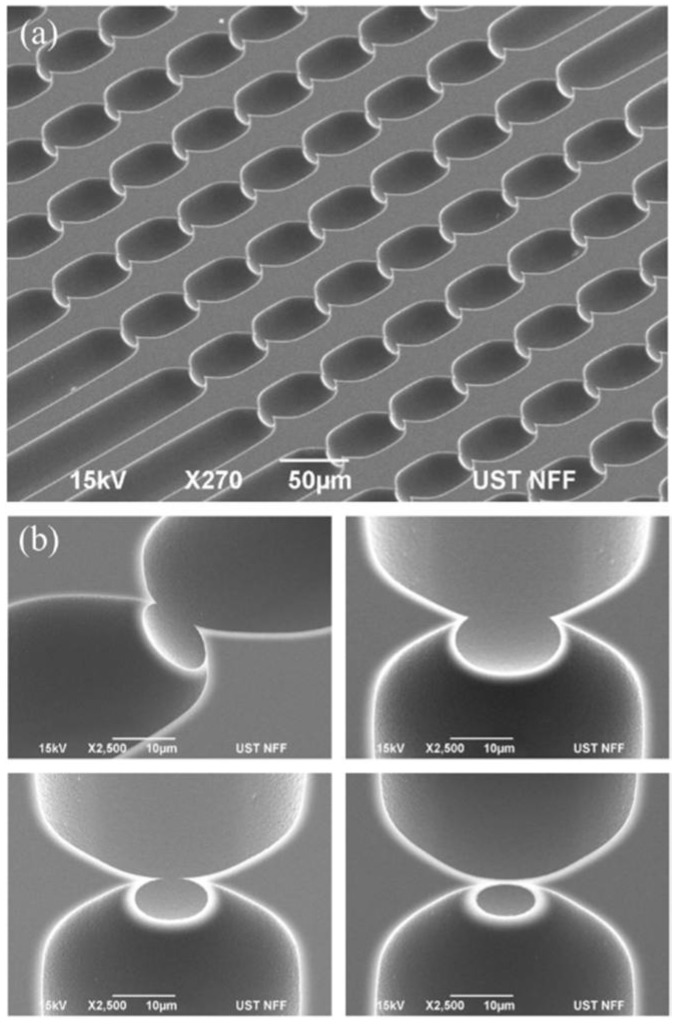
Scanning electron micrographs: (**a**) channels featuring eight single-cell point constrictions and (**b**) constrictions of various sizes in close-up views. Scale bar: (**a**) 50 μm; (**b**) 10 μm.

Figure 2a shows electron micrographs of a device section focusing on the point constrictions in silicon (before bonding the glass cover). Multiple point constrictions are arranged in cascades along straight channels, with each being 40 μm wide and 7 mm long in nominal values. The channels, 75 in total, with each featuring identical point constrictions in size and count, share the inlet and outlet ports (details in Figure S1a). Across the devices, variations mainly include the constriction count per channel and the constriction size defined in terms of the maximum width w and depth d in Figure 1b. Point constrictions are of a round geometry and the constrictions of various sizes are depicted in Figure 2b.

### 3.2. Cell Lysis

**Figure 3:**
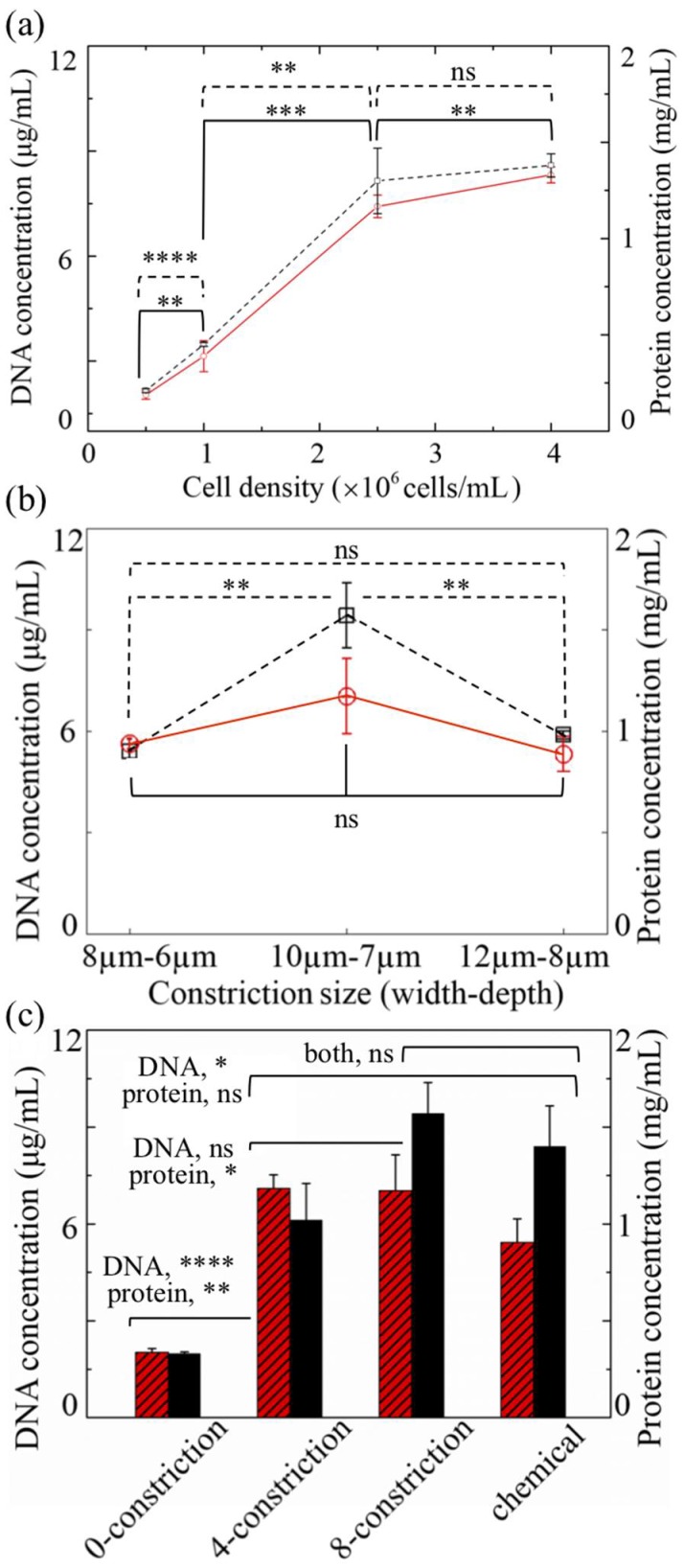
Protein (dashed lines; light-shaded columns) and DNA (solid lines; dark-shaded columns) concentrations of mechanically lysed mouse embryo fibroblast (NIH/3T3) cells obtained by the device as a function of (**a**) the cell population density, (**b**) the constriction size, and (**c**) the number of constrictions per channel. The devices in (**a**,**b**) feature eight constrictions per channel. Those in (**a**,**c**) measure w=10 μm and d=7 μm. The population density in (**b**,**c**) is 2.5 × 10^6^ cells/mL. In (**c**), the controls refer to the lysates obtained from a device without any constriction or from a conventional chemical method. (ns: not significant; * p<0.05; ** p<0.01; *** p<0.001; **** p<0.0001; two-tailed *t*-test). Error bars: ±1 SD (n=3).

NIH/3T3 cells were used for a quantitative assessment of the device performance, and the lysates were assayed for the total protein and DNA concentrations. Figure 3a shows the results for a population density of 0.5, 1, 2.5, and 4 × 10^6^ cells/mL after an eight-constriction treatment (w=10 μm, d=7 μm). It should be noted that fluid velocities and stress levels in this particular design exceed 10 m/s and 100 kPa, respectively, for the applied pressure (7 bar) according to numerical simulations (Figure S3). Both the DNA and protein yield proportionally increase with an increase in population density up to 2.5 × 10^6^ cells/mL, reaching an average of 7.4 μg/mL and 1.3 mg/mL, respectively. A further increase to 4 × 10^6^ cells/mL contributes to both the DNA and protein yield only slightly, in comparison. Since the increase in the protein yield is statistically insignificant, a cell population density of 2.5 × 10^6^ cells/mL was chosen for the subsequent experiments.

Figure 3b,c show the influence of the constriction size, as well as the constriction count per channel, respectively. A slight deviation in size, whether that be an increase (w=12 μm, d=8 μm) or a decrease (w=8 μm, d=6 μm), leads to a concentration drop in both the total protein and DNA level; however, the drop in DNA on average (from 7 to ~5 μg/mL) is statistically insignificant and less noticeable than that in the protein (from 1.6 to ~1 mg/mL) for both the constriction sizes. Through the large constriction, cells continue to deform, but probably not to the extent that can instigate their effective disruption, whereas through the small constriction, they display clogging, which limits the elution of DNA and proteins.

Figure 3c also shows the results from the controls: a conventional chemical lysis method and a treatment in an identical design, but without any constriction. As can be seen, the DNA yield and protein yield delivered by the latter group are substantially lower than those of the four- and eight-constriction treatment groups, indicating that the constrictions do indeed promote effective cell lysis. The mean DNA concentrations from the four- and eight-constriction treatment groups are comparable and hover around 7 μg/mL, exceeding the average value of the chemical group (about 5.43 μg/mL), albeit by an amount statistically insignificant for the eight-constriction treatment. Contrary to this, the mean protein concentration is noticeably lower for the four-constriction treatment (~1 mg/mL) than for the eight-constriction treatment (1.57 mg/mL) and neither treatment shows a statistically significant deviation from the chemical group (1.4 mg/mL).

About 15 μg/mL DNA and 1.25 mg/mL total protein exist within a population of 2.5 × 10^6^ cells/mL, based on the reported values for a single mammalian cell, with values of 6 and 500 pg [30,31,32,33]. These are only rough estimates because the reported figures vary according to the cell cycle and type, and the measurement method. However, the numbers are still meaningful enough to put the results into perspective. Particularly, the DNA yield of either method suggests a possible loss of DNA in the post-treatment step (centrifugation). That is, the nuclei or chromosomes being intact might have been pelleted along with the membrane debris (more on this later). Chemical lysis could also suffer from such loss, probably more so, due to the absence of proteases that digest histones, which are the proteins that hold DNA being packed within the chromosomes. Moreover, the chemical lysis buffer contains protease inhibitors added to preserve proteins, which also favor the intact chromosomes. Meanwhile, mechanical treatment could still shear at least a fraction of the chromosomes; the smaller the fragments, the more readily they escape centrifugation and stay within the lysate, unlike the intact chromosomes, which are likely to sediment. This might explain the relatively poor DNA yield of the chemical method in relation to that of the four-constriction treatment.

Previous studies on the topic have reported the concentration values for total nucleic acids, including dsDNA, ssDNA, and RNA, based on the absorbance readings at 260 nm [21,23,24]. Although this method was initially adopted here for convenience, it was found to be unreliably inaccurate and was abandoned for a fluorometric method, which is known to be more sensitive and accurate and highly specific to dsDNA [34]. Similarly, the total protein levels were measured here using a colorimetric assay (BCA) instead of absorbance levels measured at 260 and 280 nm (Warburg Christian method) [35]. While the absorbance spectrophotometry is an established technique for the rapid evaluation of protein and nucleic acid levels, its accuracy highly depends on the sample purity [36]. Pure or nearly pure samples of nucleic acids or proteins can be accurately measured contrary to their complex mixtures, which, however, reportedly give inaccurate readings [37,38,39].

### 3.3. Cell Types

Cell lysis conducted through point constrictions depends on the cell stiffness, as well as the cell size in reference to the constriction size through an intricate relation. Cells show variations in size and stiffness across distinct types to the extent that they are probably far greater than the variations expressed within a specific type. For instance, metastatic cancer cells are reportedly 70% or more softer than the benign types that line the body cavity [40]. As such, these cells are expected to be less prone to rupture under mechanical stress levels typically disruptive to benign cells. In addition to fibroblasts (NIH/3T3 cells), the two other cell types that underwent treatment here in an eight-constriction device (w=10 μm and d=7 μm) include cancerous lines (HCT116 and K562 cells).

**Figure 4:**
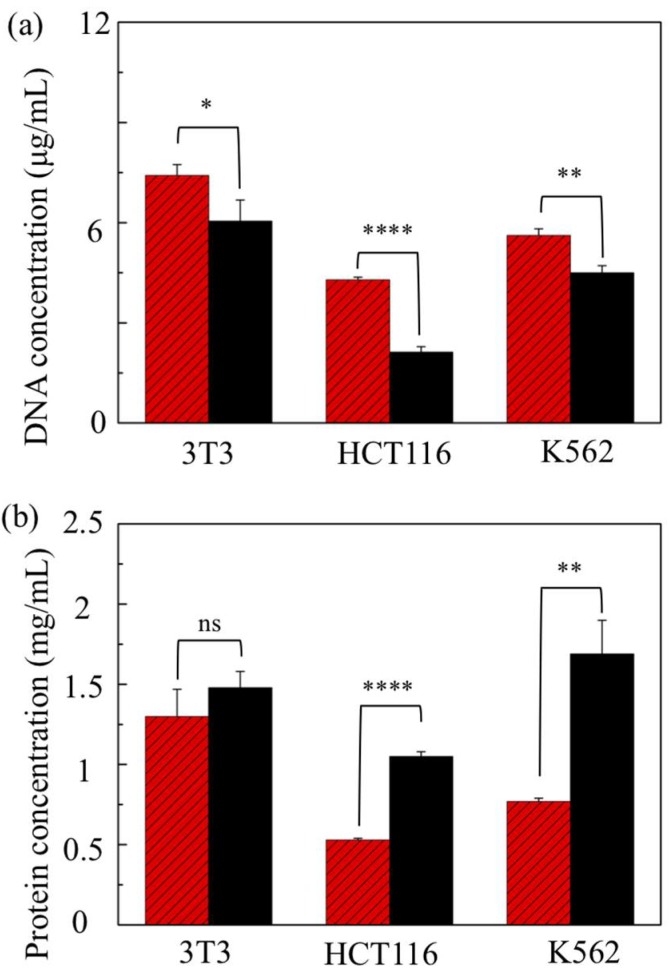
(**a**) DNA and (**b**) protein levels in the lysates of three distinct cell lines obtained through an eight-constriction treatment (light-shaded columns) and a chemical treatment (dark-shaded columns). The constrictions are w=10 μm and d=7 μm. The population density is 2.5 × 10^6^ cells/mL. (ns: not significant; * p<0.05; ** p<0.01; **** p<0.0001; two-tailed *t*-test). Error bars: ±1 SD (n=3).

Figure 4 shows the results in relation to those obtained from the conventional chemical method. The general trend is that the DNA yield is still higher for the device treatment than for the chemical method, whereas the protein yield shows the opposite trend. The output discrepancy between the two methods is strikingly large for the cancerous cell lines (both HCT116 and K562 cells) compared to the fibroblasts. In particular, the protein concentration of the device lysates for either of the cancerous lines is about half of the respective chemical lysate level. This is unlikely to be due to ineffective cell rupture because the device yields more DNA than the chemical method, noticeably for K562 cells and even more so for HCT116 cells (about a twofold increase). It could be, however, due to a loss of proteins discarded together with all the pelleted membrane fragments without further processing to minimize such loss. Since mechanical lysis does not dissolve the lipid membrane, the membrane fragments and the membrane-intact organelles remove a considerable fraction of the total proteins. Of those, the endoplasmic reticulum (ER) is the largest organelle, which is an extensive network of lipid membrane tubules and flattened sacs spread throughout the cytoplasm [41]. ER acts as a vast processing unit for transport proteins, which make up about one-third of the cell proteome [42]. Additionally, it has a much larger membrane area than the cytoplasm because of its convoluted structure; the release of its content would be less effective unless homogenized into smaller fragments. While an eight-constriction treatment is adequate for cytosol release, releasing the content of organelles calls for additional constrictions. In the case of the fibroblasts, increasing the number of constrictions (from 4 to 8) significantly enhances the mean protein yield, increasing it further to the level of the chemical method (Figure 3c). Similarly, the use of additional constrictions per channel might further boost the protein yield in the case of cancerous cell lines.

### 3.4. Nuclei Isolation

The relatively low DNA yield encountered with both the chemical and mechanical methods has raised the issue of possible nuclei loss during the post-treatment removal of membrane debris before assaying the lysates. Scrutinizing the lysates closely after device treatment, however, led to the sighting of many intact nuclei. Instead of post-treatment centrifugation, the lysates were sent to a flow cytometer for a quantitative assessment of the nuclei isolation efficiency.

**Figure 5:**
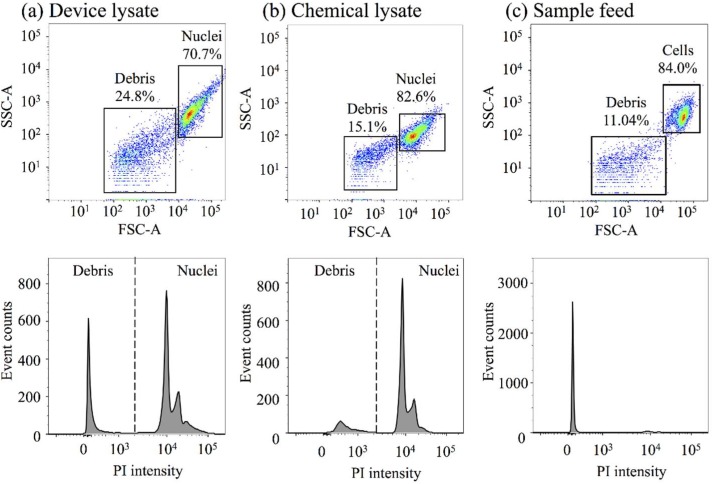
Representative scatter plots of forward-scattered (FSC) and side-scattered (SSC) data and the corresponding histograms of propidium iodide (PI) intensity from (**a**) a device lysate (from a four-constriction treatment) and (**b**) a chemical lysate, as well as (**c**) the sample feed (untreated cells). The constrictions are w=10 μm and d=7 μm.

Figure 5 shows the flow cytometric analyses of the lysates from a four-constriction treatment (w=10 μm and d=7 μm), as well as from a chemical nuclei-isolation protocol in relation to the control (the sample feed; untreated cells). For each case, the analysis involves 10,000 events. Two discrete clusters of events emerge in the dot plots of the forward-scattered (FSC, particle size) versus side-scattered (SSC, particle granularity) data. The clusters can be identified based on the particle size (high and low FSC data) and appropriately gated as the nuclei/cells (the lysates/control) and the cellular debris. A high level of debris is noted in the device lysate (~25%) in comparison to the chemical lysate (15%) and the sample feed (11%), due to the mechanical nature of the process. In contrast, the nuclei levels (PI-positive events) in the device lysate and chemical lysate appear to be comparably high, around 71% and 83%, respectively, in relation to the viable cell level in the sample feed, which is 84%. However, it should be noted that the nuclei, especially those isolated through the device, tend to form aggregates and each event might correspond to a nuclear doublet or triplet instead of a single nucleus.

**Figure 6:**
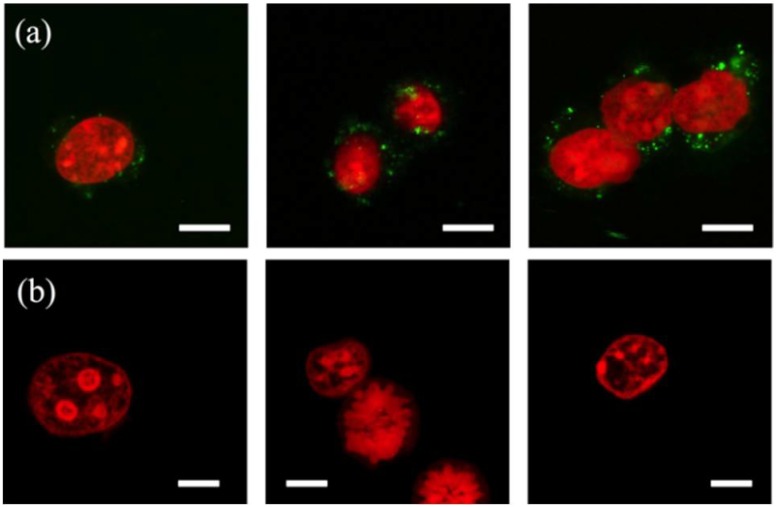
Confocal fluorescence images of the nuclei (red; propidium iodide (PI)-stained) from mouse embryo fibroblast (NIH/3T3) cells isolated using (**a**) an eight-constriction treatment and (**b**) chemical lysis. The nucleus, and the nuclear doublet and triplet shown in (**a**) carry membrane fragments (green; DiOC18(3) (DiO)-stained). The constrictions are w=10 μm and d=7 μm. Scale bars are 10 μm.

The event histograms of the lysates show a convoluted peak at a high PI intensity for the events gated as the nuclei and comprising the superposition of three constituent peaks. The peaks signify the single-nucleus events (the lowest PI intensity), and the events of nuclear doublets and triplets (the highest PI intensity), the presence of which has also been confirmed through confocal microscopy imaging (Figure 6). The nuclei isolated by either method exhibit a similar morphology although the device-isolated nuclei are not entirely segregated from the membrane fragments (DiO; green). The fragments promote aggregates, which is why the device-isolated nuclei events register a larger particle size (FSC signal) and granularity (SSC signal) than the events of the chemical lysate. These membrane fragments possibly include the remains of the nuclear envelope, a double-layered membrane with the outer layer simply being an extension of the ER membrane. Despite this physical link, the outer membrane layer contains proteins found in far higher concentrations than the ER [43], reinforcing the suggestion of protein loss during the post-treatment removal of the membrane fragments.

The intact nuclei isolation efficiency, $e_{n}$, can be estimated from the flow cytometry data according to the definition $e_{n}=\left(N/C\right)\times 100$, where N and C are the occurrence rates of nuclei and cells in the lysate and sample feed, respectively. The cell rate, C, is taken same as the event rate, assuming that each event represents a single cell, whereas the nuclei rate is the corresponding event rate, $E_{n}$, factored by the average number of nuclei per event, n: $N=nE_{n}$. The value of n is a weighted sum of the subpopulation fractions of the single-nucleus events, s, and the events of nuclear doublets, d, and triplets, t, i.e., n=s+2d+3t, all derived from the scatter plots (FSC–Height vs. FSC–Area). Table 1 lists the values derived along with the calculated values signifying the nuclei isolation efficiency. The nuclei isolation efficiency of the device emerges as 72%, whereas the efficiency of the chemical method approaches 90%. The device results are also supported by repeat experiments (Figure S4 and Table S1) and could be further enhanced by an optimized pressure and design.

## 4. Conclusions

We have demonstrated continuous-flow mechanical cell lysis and intact nuclei isolation using silicon point constrictions. Single-cell constrictions are conducive to isolating intact nuclei because of their characteristic geometry (concave nanoblade). The lysates are rich in intact nuclei, the abundance of which could be further enhanced by reducing the applied pressure, thereby avoiding any unnecessary nuclei fragmentation due to excess shear and compression. Point constrictions exert a relatively low backpressure owing to their highly localized nature, which is attractive for the manual syringe injection of cells without the requirement of a pump. The results suggest that the size and count of constrictions must be tailored according to a specific cell type for the maximum recovery of total proteins in particular. Point constrictions could be further integrated with downstream modules to access and stretch chromatins for mapping DNA, or, alternatively, to extract and detect target proteins or nucleic acids. Unlike some other nanostructures, the concave nanoblades are robust and fabricated through a low-resolution photomasking process, which is distinct from bottom-up synthesis and amenable to batch production in semiconductor foundries.

## Figures and Tables

**Table 1:** Nuclei isolation efficiency and the associated set of values derived for Figure 5.

Method	Subpopulation Fractions (%)			*n*	$E_{n}$ (s^−1^)	N (s^−1^)	C (s^−1^)	$e_{n}$ (%)
	s	d	t					
Device	83.5	10.2	6.3	1.23	133	164	227	72.24
Chemical	92.6	4.8	2.6	1.10	184	202	227	88.98

## Supplementary Materials

The following are available online at https://www.mdpi.com/2072-666X/10/7/488/s1: Figure S1: schematic for overall device layout and key process steps, Figure S2: photographs of the chip holder and experimental setup, Figure S3: numerically computed fluid velocity and stress maps, Figure S4: flow cytometry scatter plots, and Table S1: nuclei isolation efficiency and the associated set of values derived for Figure S4.
