# Supplementary material for: Single-Cell Point Constrictions for Reagent-Free High-Throughput Mechanical Lysis and Intact Nuclei Isolation
Source: Micromachines (Basel). 2019 Jul 19;10(7):488. doi: 10.3390/mi10070488 (PMC6680784; doi:10.3390/mi10070488)
Supplement: Supplementary file 1 [file micromachines-10-00488-s001.pdf]

micromachines

Supplementary Materials

Single-Cell Point Constrictions for Reagent-Free High-Throughput Mechanical Lysis and Intact Nuclei Isolation

Xiaomin Huang [1], Xiaoxing Xing [1,2], Chun Ning Ng [3] and Levent Yobas [1,3,*]

[1] Department of Electronic and Computer Engineering, The Hong Kong University of Science and Technology, Clear Water Bay, Kowloon, Hong Kong, China
[2] College of Information Science and Technology, Beijing University of Chemical Technology, Beijing 100029, China
[3] Division of Biomedical Engineering, The Hong Kong University of Science and Technology, Clear Water Bay, Kowloon, Hong Kong, China
* Correspondence: eelyobas@ust.hk

Supplementary Figures

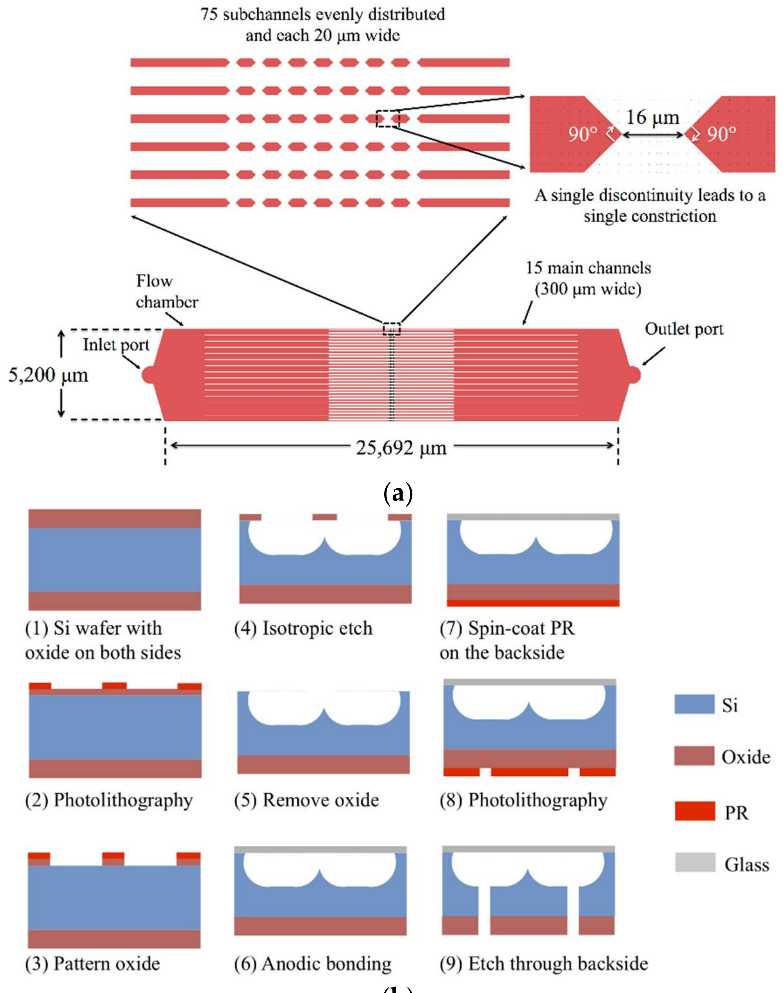

Figure S1. (**a**) Overall device layout: a cascade of eight constrictions (discontinuities) along each subchannel. Note that the channel dimensions indicated are valid for the mask layout and the final dimensions on the fabricated device are larger due to the isotropic etch undercut. (**b**) Key process steps: sketches of a device cutaway view along the longitudinal axis shown for a fluidic channel featuring a single point constriction. PR: photoresist film.

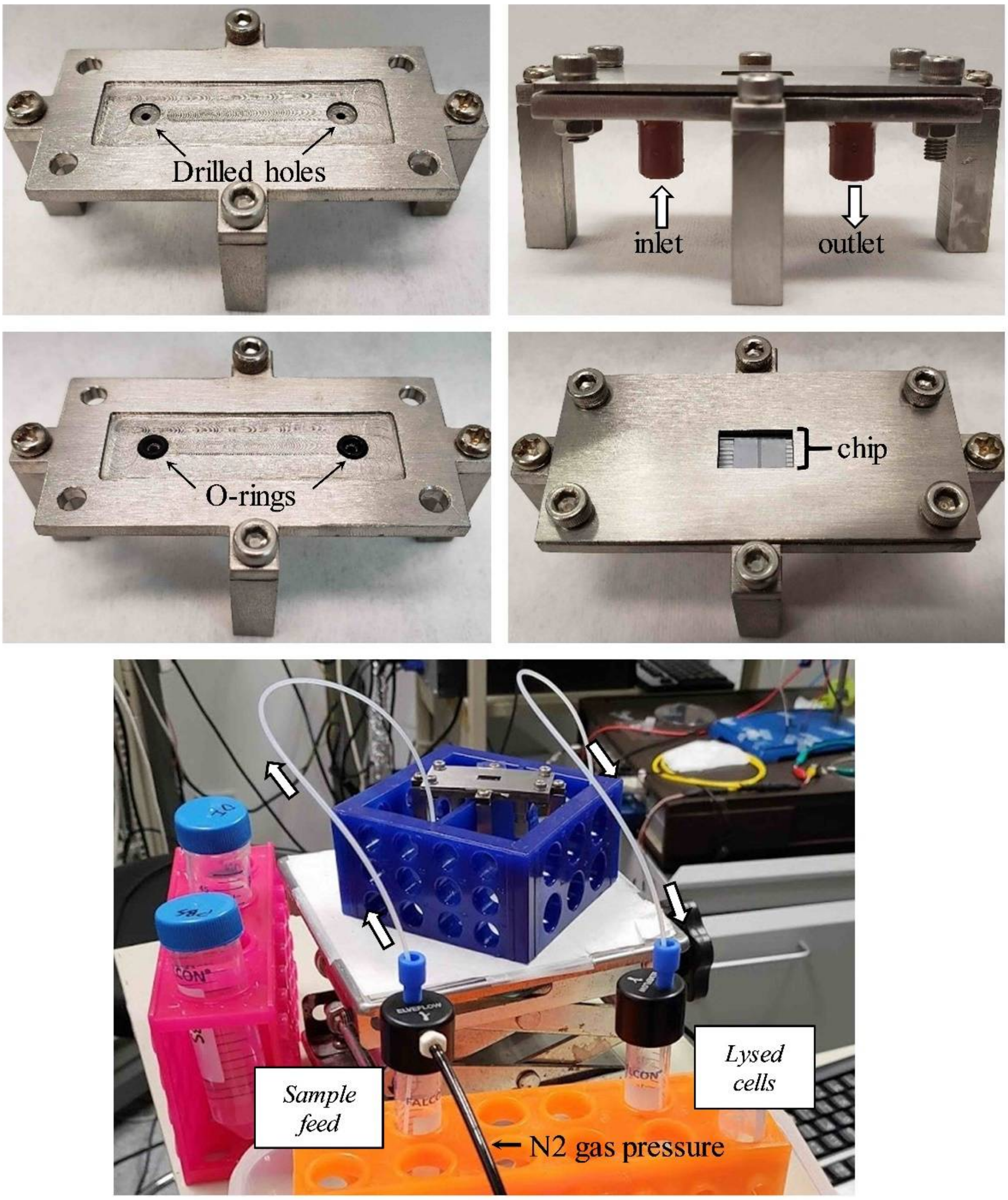

Figure S2. Photographs illustrating the custom-designed chip holder (**above**) and experiment setup (**below**).

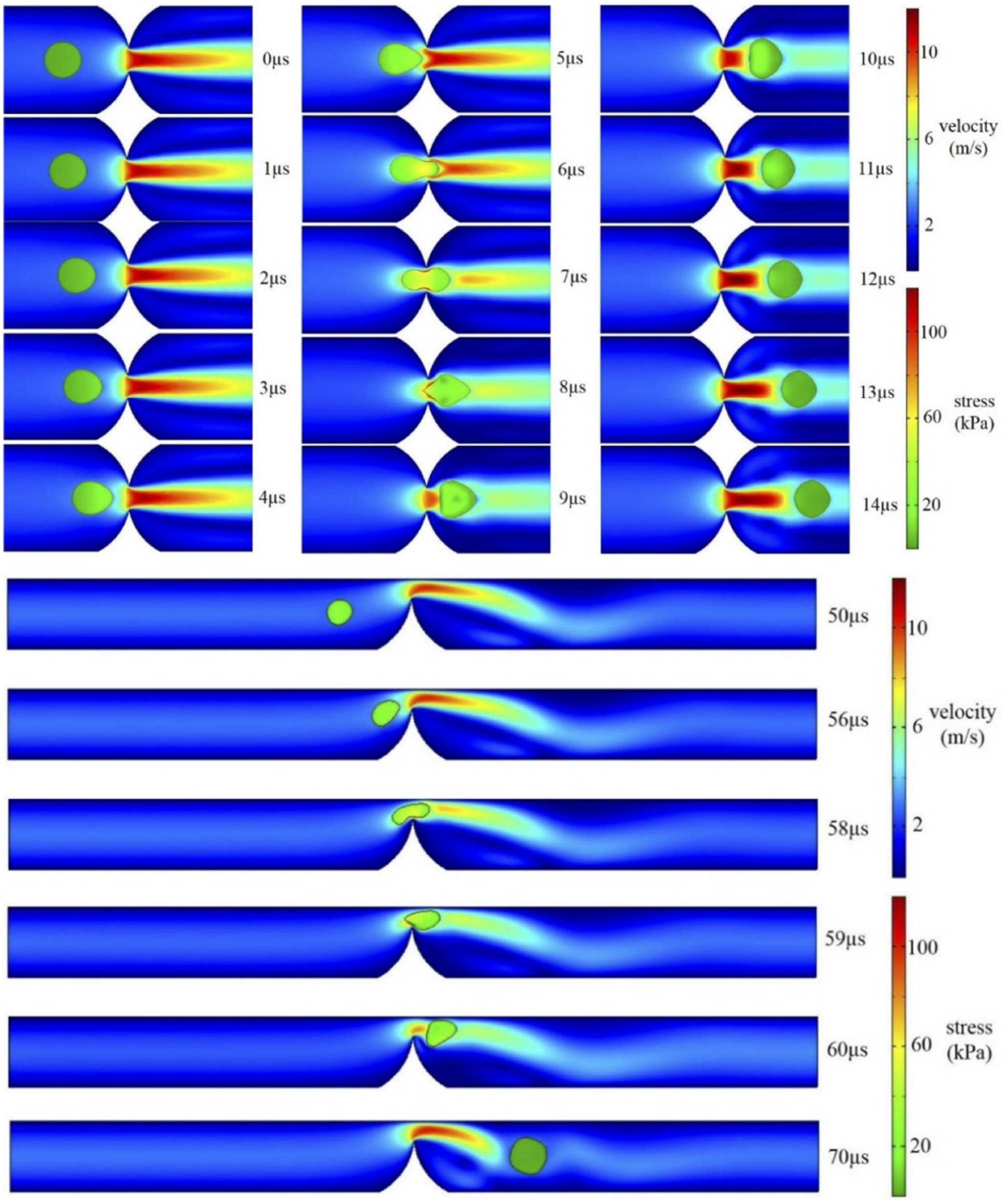

Figure S3. Numerically computed (COMSOL) fluid velocity and stress maps for a two-dimensional model: (**a**) x-y plane and (**b**) x-z plane (defined in Figure 1). Input: 0.6 bar, which is average pressure drop across a constriction in an eight-constriction design (w = 10 µm; d = 7 µm) computed for a cell-free fluid flow under the applied pressure of 7 bar [1]. Cell: size, 15 µm; Young's modulus, 97 kPa; Poisson's ratio, 0.49. Fluid: density 1000 kg/m^3; viscosity 1.01 mPa s. Channel: width, 43 µm; depth, 26 µm; length, 300 µm. Time stamps in (a) and (b) are independent and referenced to arbitrarily chosen origins.

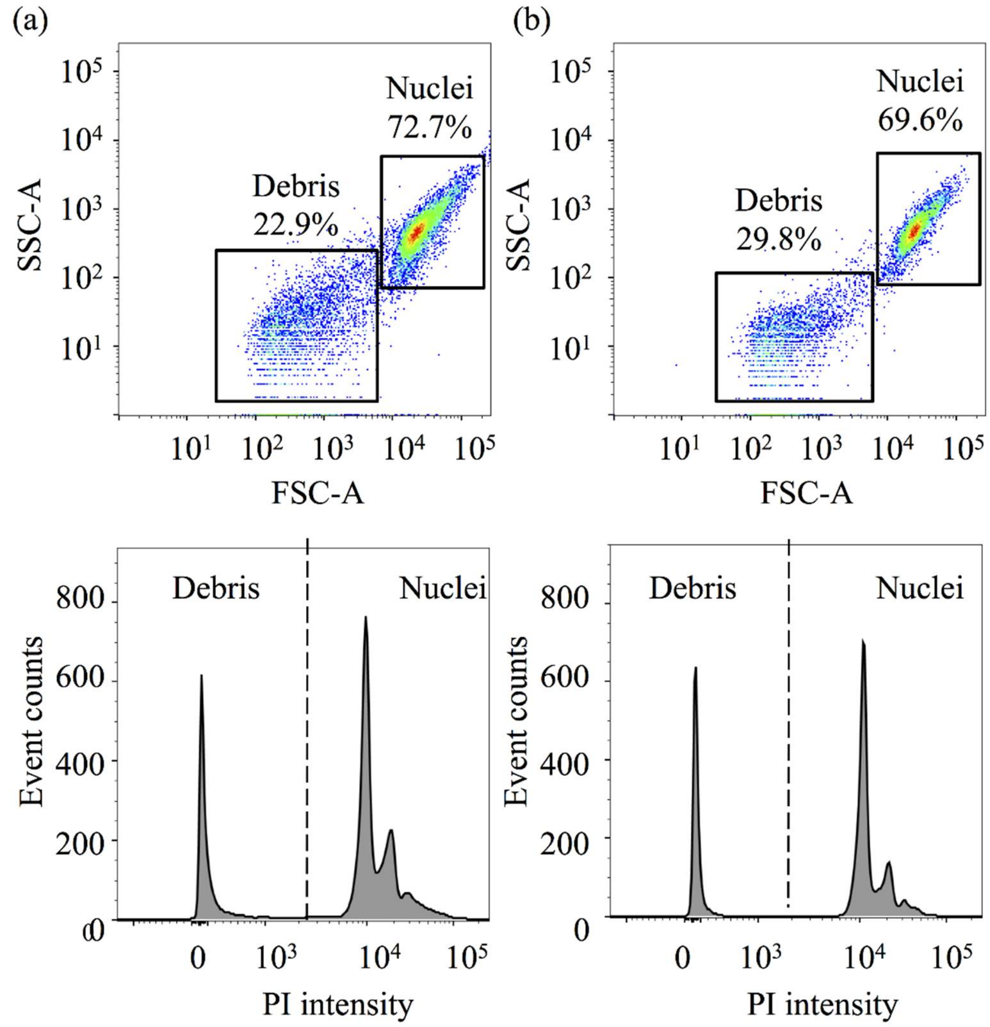

Figure S4. Representative scatter plots of FSC and SSC data and the corresponding histograms of PI intensity of device lysates obtained from a four-constriction treatment in separate but identical devices. The constriction size: w = 10 μm and d = 7 μm.

Supplementary Tables

Table S1. The nuclei isolation efficiency and the associated set of values derived for Figure S4.

Experiment	Subpopulation fractions (%)			n	E_n (s^{-1})	N (s^{-1})	$C^{\#}$ (s^{-1})	e_n (%)
	s	d	t					
Figure S4a	84.7	6.9	8.4	1.24	127	158	256	61.72
Figure S4b	84.2	11.5	4.3	1.20	136	163	227	71.80

$^{\#}$From two separate sample feeds

References

1. Huang, X. Reagentless Mechanical Lysis for Cell Contents Extraction through Microfluidic Constrictions. M.Phil. Thesis, The Hong Kong University of Science and Technology, Hong Kong, SAR, 2018.
